# Fabrication of Graphoepitaxial Gate-All-Around Si Circuitry Patterned Nanowire Arrays Using Block Copolymer Assisted Hard Mask Approach

**DOI:** 10.1021/acsnano.0c09232

**Published:** 2021-05-27

**Authors:** Tandra Ghoshal, Ramsankar Senthamaraikannan, Matthew T. Shaw, Ross Lundy, Andrew Selkirk, Michael A. Morris

**Affiliations:** † School of Chemistry, AMBER and CRANN, Trinity College Dublin, Dublin, Ireland D02 AK60; ‡ Intel Ireland Ltd., Collinstown Industrial Park, Leixlip, Co. Kildare, Ireland W23 CX68

**Keywords:** block copolymer, graphoepitaxy, hard mask, nanowire, gate-all-around

## Abstract

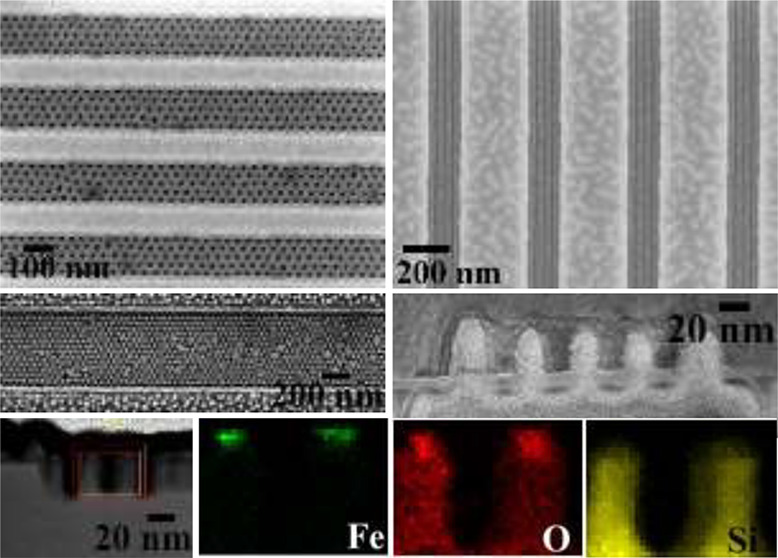

We demonstrate the fabrication of sub-20 nm gate-all-around silicon (Si) nanowire field effect transistor structures using self-assembly. To create nanopatterned Si feature arrays, a block-copolymer-assisted hard mask approach was utilized using a topographically patterned substrate with well-defined Si_3_N_4_ features for graphoepitaxially alignment of the self-assembled patterns. Microphase-separated long-range ordered polystyrene-*b*-poly(ethylene oxide) (PS-*b*-PEO) block-copolymer-derived dot and line nanopatterns were achieved by a thermo-solvent approach within the substrate topographically defined channels of various widths and lengths. Solvent annealing parameters (temperature, annealing time, *etc*.) were varied to achieve the desired patterns. The BCP structures were modified by anhydrous ethanol to facilitate insertion of iron oxide features within the graphoepitaxial trenches that maintained the parent BCP arrangements. Vertical and horizontal ordered Si nanowire structures within trenches were fabricated using the iron oxide features as hard masks in an inductively coupled plasma (ICP) etch process. Cross-sectional micrographs depict wires of persistent width and flat sidewalls indicating the effectiveness of the mask. The aspect ratios could be varied by varying etch times. The sharp boundaries between the transistor components was also examined through the elemental mapping.

The gate-all-around silicon nanowire field effect transistor is a high-performing nanoscale device having superior electrical performance to gate on top structures in terms of low leakage, short channels, and high drive current due to greater effective mass and density of states within a channel.^1−3^ In the modern microelectronics industry the economies of scale and rapid decreases in transistor dimension are limited by extensions of conventional lithography.^1−3^ According to current expectations, ArF immersion with multiple patterning and EUV lithography are the most likely routes to sub-7 nm FINFET technologies (before translating to revolutionary device architectures) but are limited by persistent delays in high throughput, production cost, and unresolved mask defectivity issues.^4−6^ Thus, innovative cost-effective technological approaches are needed to reach the critical resolution and lower line edge roughness (LER) of the transistor structures whose performance is critically dependent on high quality and size regularity of the devices. To achieve improved complementary metal oxide semiconductor (CMOS) applications, a high density of nanoscale transistors must to be realized.^7,8^ Among different nanofabrication bottom-up methods, directed self-assembly (DSA) of microphase-separated structures of BCPs is considered favorable for its high throughput, low cost, and simplicity of generating high density sub-10 nm contact, line, and space shaped patterns on substrates.^9−12^ However, due to large defect annihilation energies, defect-free self-assembled BCP films cannot be achieved without enabling technologies. Lithographically defined guiding patterns are used to produce long-range ordered arrangements of BCPs via chemoepitaxy^13,14^ or graphoepitaxy.^15−17^ In the latter approach, topographically defined features align the BCP patterns by physical and chemical confinement and result in low defect densities.^18,19^ Within these topographies, BCP film thickness variation can be observed by following spin coating depending on the dimension and density of the guiding pattern. This can lead to the formation of either a thicker islands of patterns and matrix edges or a thinner film over the dense features.^20−22^ In order to overcome these critical issues, the polymer-solution concentration and the depth of the guiding features (trench depth) need to be optimized avoiding overfilling or underfilling (noting that solvent swelling during film processing can be important).^23,24^ Even with the need for alignment of the pattern, methodology is potentially important for semiconductor manufacturing as significant capital investment (compared to top-down lithographies) is not required as the minimum printable pitch is solely determined by the BCP.^25^

In simple BCP lithography, selective removal of one of the blocks generates the template (mask) for pattern transfer onto the substrate. To fulfill the manufacturing requirements of low defectivity and LER as well as controlling the microdomain orientation (vertical or parallel to the substrate plane) of the BCP nanostructures strict control and understanding of film thickness, block composition, and in particular, interfacial energies of the film and the substrate as well as the air–surface interface is needed.^26,27^ Cylinder forming BCP systems are simpler to control compared to lamellar systems since interfacial energy control is less challenging.^28^ However, the pattern transfer of the cylindrical phase BCP system is complicated due to obstruction of the blocks inside the matrix and additionally, the circular cross-section of the cylinders leads to shaped features due to etch selectivity.^29−31^ A good quality pattern transfer is defined by uniform, ordered, and well-defined surface features which depend on the block selectivity and directionality of the etching protocol and reproducibility. Hard masks are generally preferred compared to soft polymer material masks because of higher etch resistivity compared to silicon. Different hard masks such as dielectrics (SiO_2_, Al_2_O_3_, and Si_3_N_4_), metal oxides, and metals have been used.^32−35^ Hard masks are also required to ensure high aspect ratio of features and simple processing routes to these are required.^36,37^ This report is of significance combing simplicity, controllability, and cost using self-assembly with an “*in situ”* hard mask technique.^38−41^

In our previous reports,^40,41^ good sidewall profile, high aspect ratio Si nanofeatures were achieved by “*in situ*” inclusion of iron oxide as a hard mask. In this report, we have explore the methodology further to achieve long-range, well-ordered vertical and horizontal Si nanowires through graphoepitaxy of SiO_2_/Si trenches with Si_3_N_4_ sidewall toward gate-all-around application. The morphology and orientation of BCP templates were controlled by a solvent annealing approach using different annealing solvents, temperature, *etc*. The morphology and interfaces of the Si nanostructures within trenches were systematically studied using microscopy and elemental mapping.

## Results and Discussion

### Schematic Description of the Si Nanopatterns Fabrication-Process Flow

**Scheme 1:**
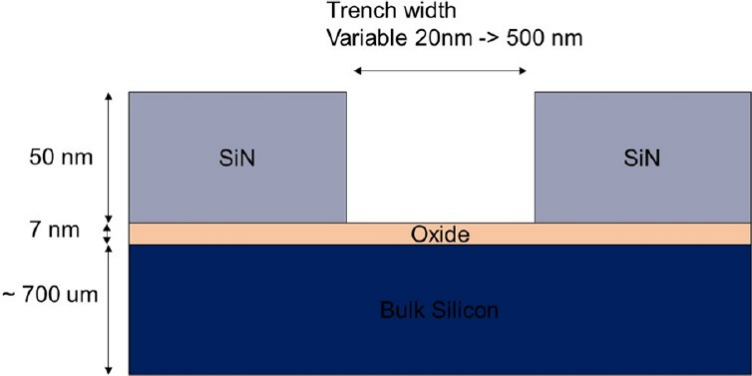
Design of the Graphoepitaxial Substrate

**Scheme 2:**
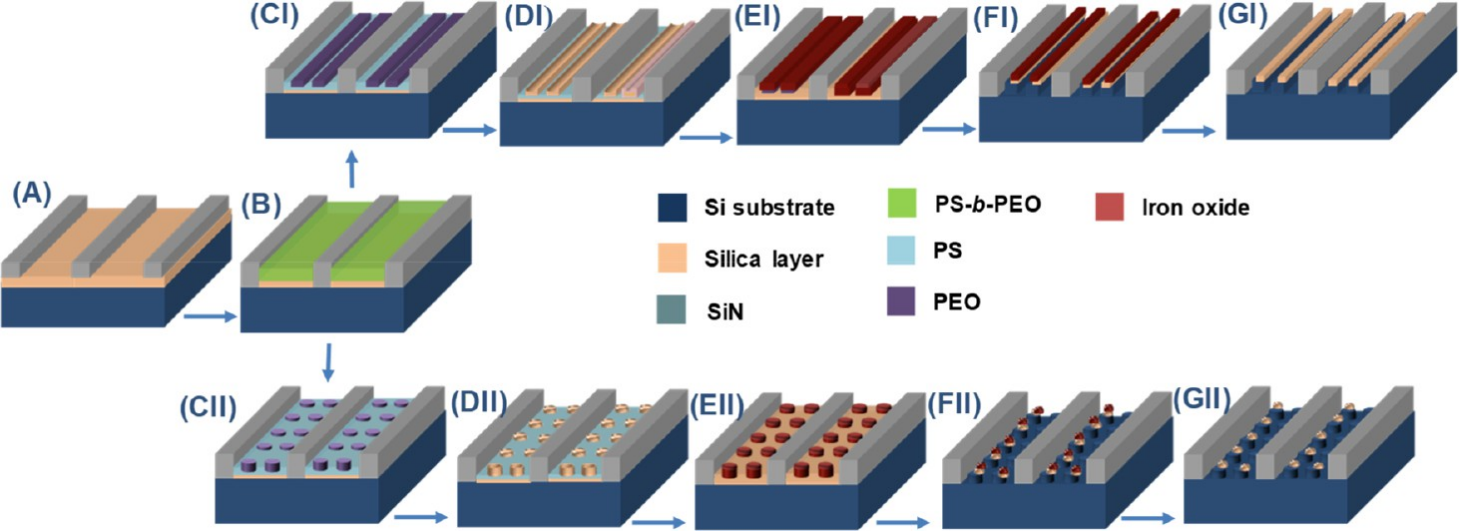
Processing Steps for Fabricating Si Nanowires Arrays within the Trenches (A) Graphoepitaxial substrate with channels of 7 nm silica-coated Si and SiN top sidewalls. (B) Spin-coated BCP film within channel. Hexagonally arranged PEO cylinders (CI) parallel and (CII) perpendicular to the substrate in PS matrix after solvent annealing. (DI and II) Modification of PEO cylinders creates nanoporous templates for the metal ion inclusion. Iron oxide horizontal (EI) and vertical (EII) nanowires were prepared by spin coating a metal ion precursor solution followed by UV/ozone treatment. Horizontal (FI) and vertical (FII) Si nanowires with iron oxide at the top by consecutive silica and silicon ICP etch. Horizontal (GI) and vertical (GII) Si nanowires with a silica layer at top after removal of oxide masks.

Scheme 1 describes the design of the graphoepitaxial substrate used for this study. The substrate is silicon with a 7 nm thick passive silica coating with 50 nm deep topographically defined patterns of Si_3_N_4_ above the passive film. The trench widths varied from 20 to 500 nm. A schematic illustration for the fabrication of the Si nanowires within the trenches is shown in Scheme 2. The substrate (Scheme 2A) was spin-coated with an appropriate amount of BCP solution (Scheme 2B). Annealing of the spin-coated film in toluene at different processing parameters produces hexagonally arranged PEO cylinders either parallel (Scheme 2CI) or perpendicular (Scheme 2CII) to the substrate in a PS matrix. Etch/modification of PEO cylinders generates nanoporous templates for metal ion inclusion (Scheme 2DI and II). Iron oxide horizontal (Scheme 2EI) and vertical (Scheme 2EII) nanowires were prepared by insertion of metal ions by spin coating the precursor solution following oxidation and polymer removal by UV/ozone treatment. Horizontal (Scheme 2FI) and vertical (Scheme 2FII) Si nanowires with iron oxide at the tip were fabricated by silica followed by Si ICP etch processes. Horizontal (Scheme 2GI) and vertical (Scheme 2GII) Si nanowires with a silica layer at the top were fabricated after removal of oxide masks.

### Formation of Hole/Space BCP Nanopatterns within the Trench

**Figure 1:**
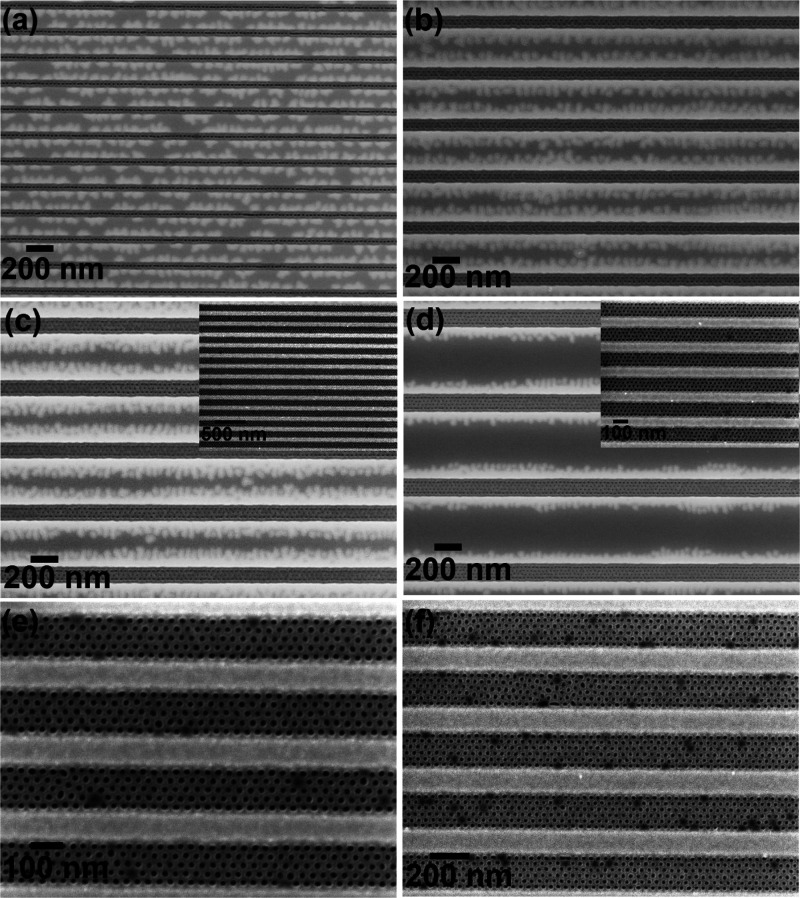
Arrays of BCP (42k–11.5k) dot patterns within trenches of the SiN substrate solvent annealed in toluene at 50 °C for 1 h with trench widths of (a) 45 nm, (b) 90 nm, (c) 135 nm, (d) 180 nm, (e) 225 nm, and (f) 270 nm, respectively.

In DSA graphoepitaxy, the topography of the substrate can affect the orientation of the BCP microdomains. A cylinder-forming hexagonal phase PS-*b*-PEO (42k–11.5k) BCP was used to achieve hole/space and line/space nanopatterns within trenches. No pretreatment of the substrate was necessary to generate the patterns. This was further confirmed by a neutral PS brush layer deposition within trenches before spin coating the polymer–toluene, solution and the results are described in the Supporting Information. To obtain highly ordered periodic BCP nanopatterns of low defectivity in the topographical features, it is essential to ensure the polymeric film fills the topography before and after solvent annealing. Thus, BCP concentrations in toluene were carefully varied to achieve well-ordered nanostructures without any overfilling/missing pattern and a uniform film thickness. BCP (0.4 wt %) was found to be the optimum concentration for pattern formation. Higher concentrations cause overfilling and surface undulation disrupting the pattern alignment while lower concentrations lead to missing regions or island formation (Supporting Information). Solvent annealing of the spin-coated films was achieved using toluene vapor at optimum temperature. Figure 1 shows SEM images of solvent annealed PS-*b*-PEO thin films annealed at 50 °C for 1 h forming hexagonal ordered PEO microdomains perpendicular to the substrate surface with a center-to-center spacing of 42 nm. Note that all the films were dipped in ethanol for 15 h at 40 °C for the modification/etching of PEO domains to enhance the electrical contrast for imaging as previously described by us.^38−41^ The brighter and darker areas correspond to PS and PEO, respectively, and result from their different mechanical properties.^38−41^ The thickness of the BCP film on a flat substrate surface was around 40 nm as measured using optical ellipsometry, and AFM suggests a similar thickness within the topographic features. Note that the guiding patterns of widths less than the BCP feature size are overfilled without any pattern being formed. As expected, the number of hexagonally long-range ordered arrays of hole/space patterns depends on the channel width. For example, 1, 2, 3, 4, 5, and 6 feature arrays of perpendicularly oriented PEO microdomains were realized (at wafer scale) for channel widths of 45, 90, 135, 180, 225, and 270 nm, respectively, without any elaborate process optimization (Figure 1). We assume that the polymeric film is entirely restrained within the topographic trenches as discontinuity is observed between adjacent sidewalls of the trenches, as seen in all recorded images.

### Formation of Line/Space BCP Nanopatterns and Effects of Annealing Temperature

**Figure 2:**
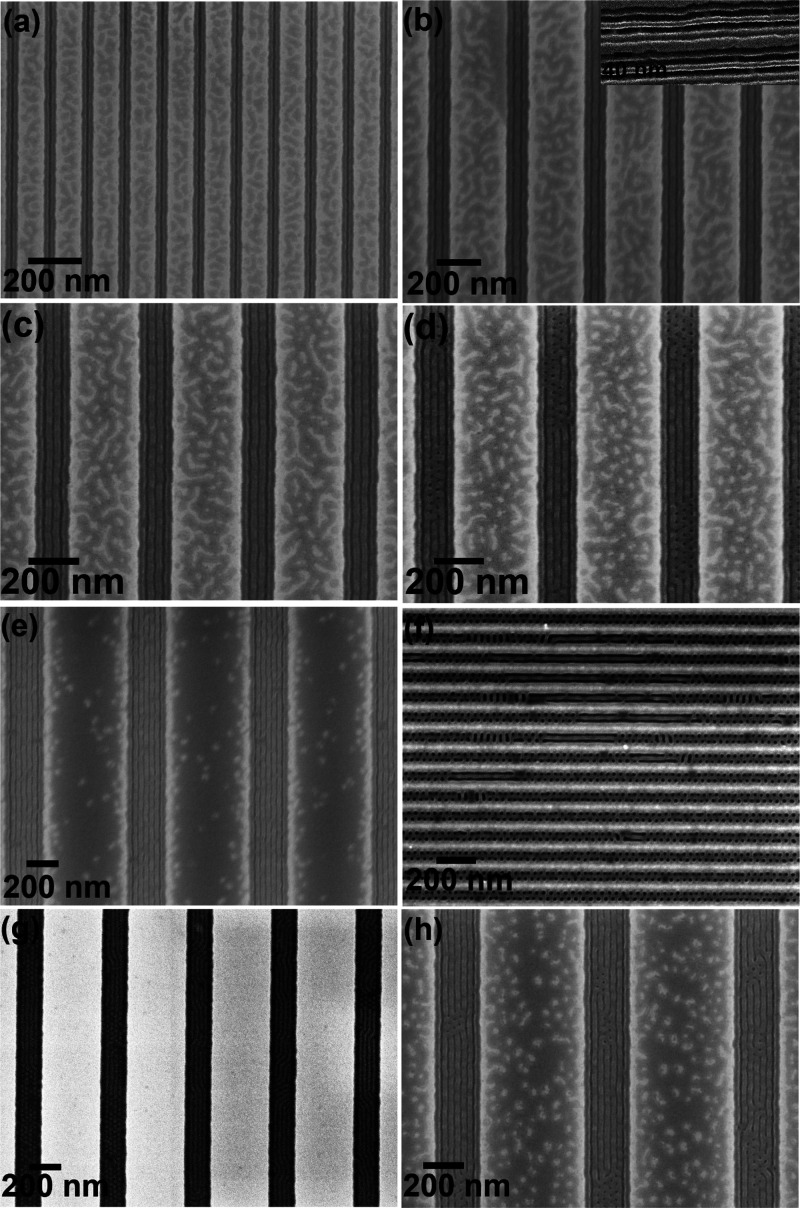
Arrays of BCP (42k–11.5k) patterns within trenches of SiN substrate solvent annealed in toluene at 60 °C for 1 h with trench widths of (a) 45 nm, (b) 90 nm, (c) 135 nm, (d) 180 nm, and (e) 270 nm, respectively. Mixture of dots and line patterns of BCP solvent annealed in toluene at 60 °C for different times of (f) 1 h 30 min, (g) 2 h, and (h) 3 h, respectively.

To inspect the effects of annealing temperature and to achieve horizontally aligned cylindrical domains, the annealing temperature was set to 60 °C, higher than the PEO block melting temperature (55 °C) for the BCP used. Figure 2a–e shows SEM images of solvent annealed PS-*b*-PEO thin films annealed at this temperature for 1 h forming hexagonally packed PEO microdomains. Arrays of 1, 2, 3, and 6 ordered horizontally oriented PEO microdomains were realized for the channel widths of 45, 90, 135, and 270 nm, respectively. Both perpendicular and parallel orientation of the cylinders were observed for the trench width of 180 nm, suggesting a complex relationship of BCP feature size to trench width. The center-to-center spacing of 42 nm and PEO cylinder diameter is around 19 nm, as measured by the magnified SEM image shown in the inset of Figure 2b. No variations in the PEO diameters were observed for the various channel widths. The line patterns extend through the entire channel length. A mixture of perpendicular and parallel orientation of cylinders (dots and lines) were observed for all the trench widths for the BCP films annealed for longer times (Figure 2f–h).

### Formation Mechanism of BCP Nanopatterns within the Trench

A number of experimental parameters influence the orientation of cylindrical microdomains including the annealing temperature, time, solvent type as well as the correlation between film thickness and surface wetting individualities. The calculated χ value of the PS-*b*-PEO system used in this study is 41.5, *i.e.*, ≫10.5 needed for microphase separation. For the PS-*b*-PEO systems, the constituent blocks exhibit asymmetric affinities for the solid substrate and the air interface. Due to favorable PEO–substrate interactions and the hydrophilic nature of PEO, this block wets the substrate and sidewalls while PS segregates to the air interface forming a PS-rich layer (the surface energy of PS is lower than that of PEO, γ_PS_ = 33 mN m^–1^; γ_PEO_ = 43 mN m^–1^).^42^ Toluene is a selective solvent for PS because of smaller solubility parameter difference with PS (δ_Tol_ – δ_PS_ = 18.3 – 18 = 0.3 MPa^1/2^) than PEO (δ_Tol_ – δ_PEO_ = 18.3 – 20.2 = 1.9 MPa^1/2^). The film swells, and there is an increase in the thickness of BCP films across the trench pattern, namely, at the center of the trench and near the sidewalls, due to exposure and absorption of toluene at an elevated temperature. A constant thickness was attained due to evaporation of the trapped solvent when samples removed from the solvent atmosphere due to rapid quenching. For an annealing temperature of 50 °C, a vertical orientation is achieved presumably due to a lower entropically hindered microphase separation as the length of PEO cylinders is similar to the film thickness. However, at extended periods or at a higher temperature of 60 °C, the cylinders can be relatively easily flipped into a parallel orientation that we believe is a result of PS segregation leading to film where the majority surface block is PS, which minimizes the free energy of the system. This is consistent with the PEO being in a semimolten state during annealing at 60 °C, allowing cylinder reorientation and segregation with little kinetic limitations. From these results, it is clear that the alignment and orientation of the domains within the trenches are dictated by two parameters (i) the complex interplay of interfacial energies between the BCP and the trench sidewalls (*i.e.*, surfaces less preferential to one of the blocks provide better guiding effect) and (ii) the depths and widths of the trenches (*i.e.*, if they are incommensurate (not close to thickness and center-to-center spacing of the BCP nanopatterns)).

### Formation of Iron Oxide Nanodots and Nanowire Arrays

Similarly ordered oxide nanodots and nanowire arrays can be generated by the metal inclusion method through spin coating the precursor–ethanolic solution followed by UV/ozone treatment as in our previous work^40^ where iron oxide nanodots were used as a resistant mask to fabricate high aspect ratio nanopatterns. The same strategy is adopted to produce silicon nanowire arrays within the trench by use of the oxide nanodots and nanowires as a hard mask.

**Figure 3:**
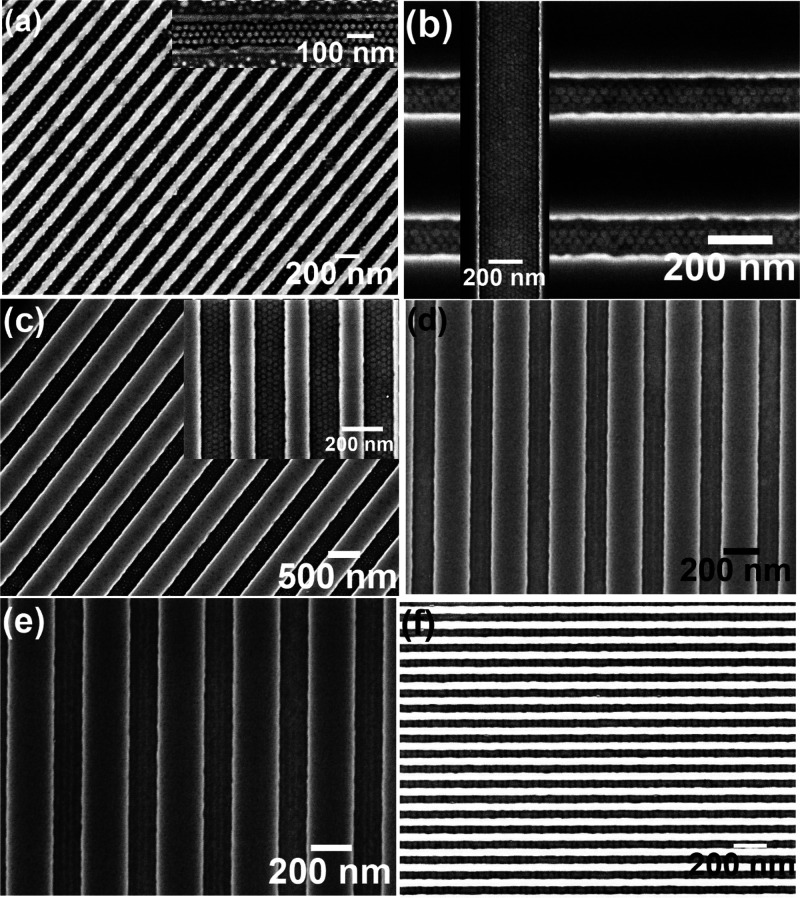
Iron oxide (a,b, c) dot patterns and (d, e, f) line patterns using different kinds of BCP templates through the inclusion method.

Figure 3 represents the SEM images of the iron oxide nanodots and lines arrays prepared using the BCP templates and an optimum amount of precursor-ethanolic solutions (0.4 wt % of iron nitrate) which was carefully controlled to generate continuous oxide dot and line features within trenches. Figure 3a–c shows arrays of dot patterns within different trench widths. The average diameter of the nanodot is 20 nm along the entire length of the trench. Arrays of 1, 3, 5 and multiple arrays of iron oxide dots were realized, as shown in Figure 3a–c and insets. As shown in the higher magnification image in the inset of Figure 3a, a darker contrast was seen alongside the trench wall, revealing the PS matrix mainly wets the trench walls. The affinity of PEO with the ionic ethanol solution favored the metal ion inclusion into the nanoporous template. The formation of a thin PEO–ethanol layer also facilitates the metal ion inclusion process via either intra- or intermolecular coordination and electron transfer from the PEO block to oxygen species in the ethanol molecule.^39,41,42^

For the line patterns, arrays of 3 and 4 nanowires are seen throughout the entire length of the trenches for 2 wt % of the iron nitrate–ethanolic solution, as shown in Figure 3d,e, respectively. Continuous and uniform diameter nanowires were formed in most of the areas within the trenches although disconnected and/or missing patterns were also evident in places. The nanowires are of uniform diameters of 16 nm along their length. Figure 3f shows nanowires across the width of the trenches with diameters of 20 nm. For all the patterns, the center to center spacing remained unchanged (42 nm as previous). The nanodots and nanowires formed using this method are physically and thermally attached firmly to the substrate. All the metal oxide nanopatterns were mimicking the polymer template patterns.

### Fabrication of Si Circuitry Nanopatterns

**Figure 4:**
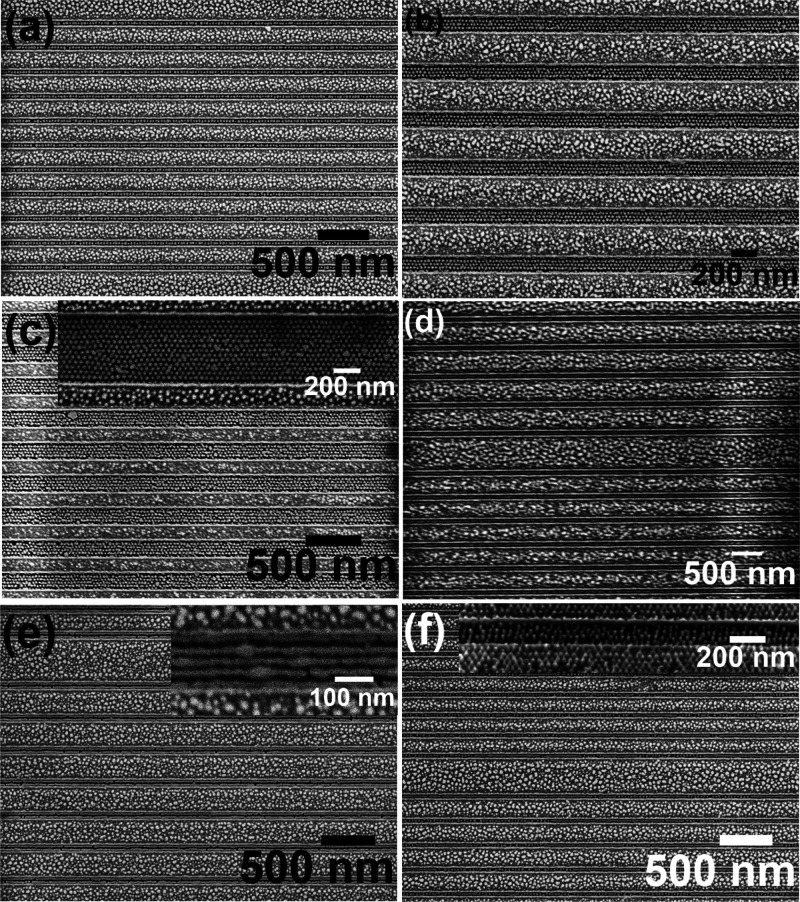
Si (a,b, c) vertical and (d, e) horizontal nanowires and (f) vertical and horizontal nanowires patterns together after pattern transfer using iron oxide as a hard mask.

To create vertically and horizontally ordered Si nanowire structures within a trench, the iron oxide nanodots and nanowires were used as a hard mask through the ICP etch process. Iron oxide-free Si nanowire arrays are obtained by treating the patterned substrate with a mild oxalic acid aqueous solution. Figure 4 shows SEM images of Si nanowire arrays over large trench areas following a 1 min Si etch. Figure 4a–c and the inset of Figure 4c show 1, 3, 4, and multiple vertical Si nanowire pattern arrays, respectively. A significant change in the SEM contrast reveals substrate etching. Moreover, the diameters and center-to-center spacing remained unchanged. This confirms the original “mask” was not significantly damaged by the etching. The efficiency of the iron oxide mask for the formation of Si nanowires verifies previous reports.^40,41^ The higher magnification image in the inset of Figure 4c shows two nanowires were fused together in a few places. This is due to “clumping” occurring during the formation of nanodots. Though the concentrations of the precursor solution was carefully optimized to create uniform monodispersed nanodots, agglomerated nanodots formed in a few places and might be due to overfilling of the precursor to some extent. Oxidization of the precursors under UV/ozone treatment leads to these agglomerated nanodots.^38^ Similarly, 1, 2, and 3 horizontal Si nanowire pattern arrays were realized following a 30 s Si etch as shown in Figure 4d,e and the inset of Figure 4e, respectively. A higher magnification image (inset of Figure 4e) demonstrates that the majority of the nanowires are continuous and of consistent diameter (15 nm) along the length although few broken nanowires can be seen with enlarged diameter sparingly observed. The center to center nanowire spacing remained unchanged. A similar etching protocol was applied for the mixed morphological oxide mask patterns, and Figure 4f reveals ordered vertical and horizontal Si nanowire patterns within the same trench. The inset of Figure 4f shows cross-sectional tilted SEM image of the vertical Si nanowires showing effective pattern transfer to a constant depth.

### Determination of Aspect Ratio Variation by Cross-Sectional TEM

**Figure 5:**
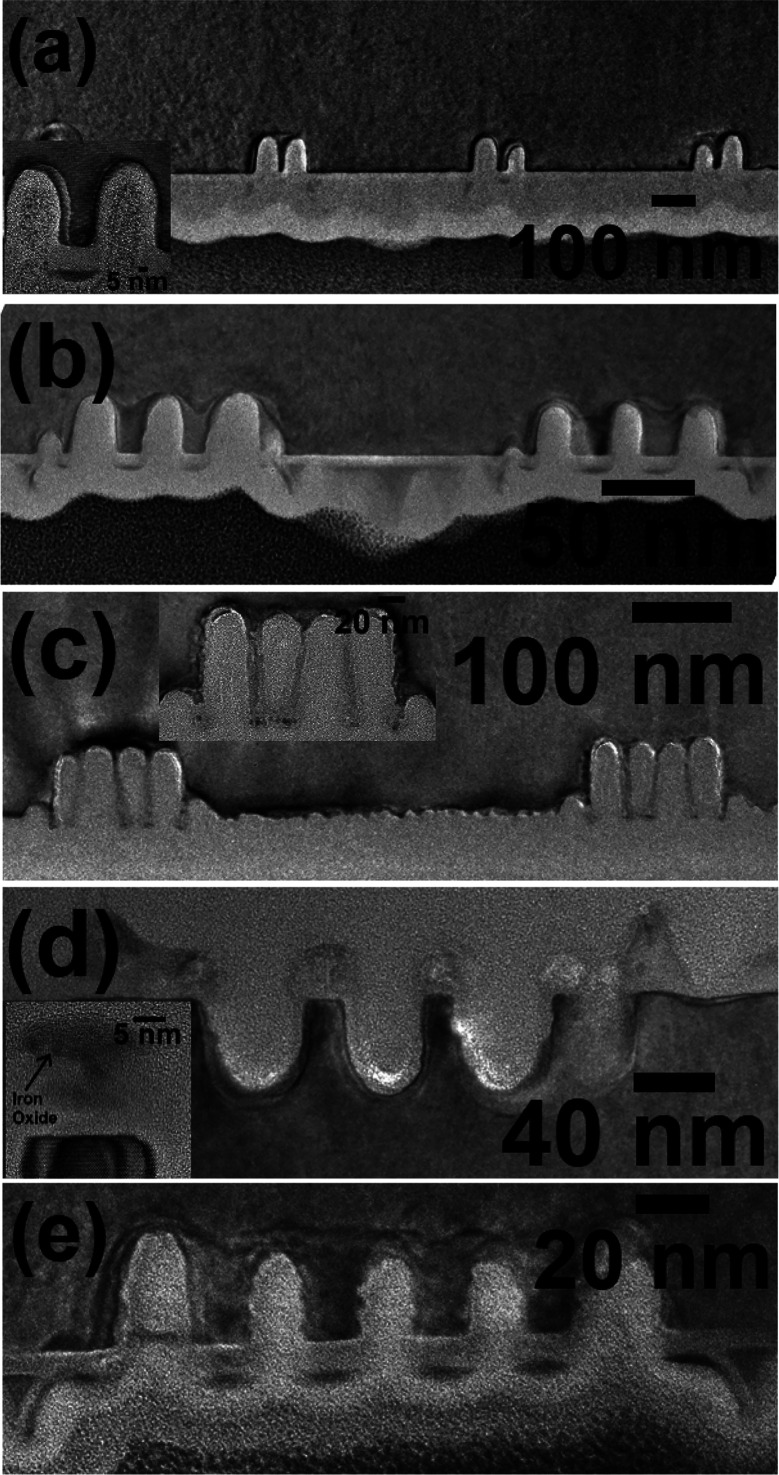
FIB cross sectioned TEM images of Si (a, b, c) vertical and (d, e) horizontal nanowire patterns with iron oxide at top for different etch times of (a, b, d, e) 30 s and (c) 1 min.

The aspect ratio of the vertical and horizontal nanowires within the trenches can be varied by varying the Si etch time. Figure 5a–e represents the FIB cross-sectional TEM images of the Si nanowire arrays with iron oxide at top following etches of 30 and 60 s. All the wires are of constant width and flat sidewalls signifying the effectiveness of the mask. Figure 5a–c represents 1, 4, and 5 arrays of vertical Si nanowires with iron oxide at the top within different trench widths. The images reveal nanowires of diameter about 20 nm with the expected 42 nm spacing. For the etch time of 30 s, 50 nm long vertical nanowire can be fabricated, as shown in Figure 5a,b. Figure 5b shows two vertical nanowires at the center and two nanowires near the sidewalls of the trenches. Compared to the vertical sidewall Si nanowires, central wires are longer because of the shallow nature (not rectangular) of the sidewalls. Figure 5c reveals 5 arrays of 100 nm long nanowires. Similar etch depths of 50 and 100 nm were realized for the horizontal nanowire arrays within trenches for the etch time of 30 and 60 s, respectively (Figure 5d,e). All the wires examined had a rectangular outline with even sidewalls and small variations of wire width along the length. The diameter of the nanowire is about 16 nm. The inset of Figure 5d shows 5 nm thick iron oxide nanowire at the top of the Si wire. Notably, the nanowires remained unaltered by iron oxide removal. From the device fabrication, it is necessary to remove the iron oxide from the top of the nanowires. After eliminating the thin iron oxide layer, Si nanowires with a top silica layer are obtained. Further, the ordered arrangement of the patterns was not affected neither by the etching process nor by the oxide removal. The structural integrity and the effectiveness of the mask during the pattern transfer process is revealed by the cross-sectional images. No surface roughening or pattern damage is realized with increasing height of the nanoarrays. Thus, high aspect ratio vertical and horizontal silicon nanowires with a controlled placement, diameter, and spacing within the trench can be realized by using iron oxide as a hard mask using ICP etching. Additionally, mask-free Si nanofeatures can be realized without any pattern damage, avoiding innovative and complicated processing steps.^40^

### Elemental Interfaces by EDX Mapping

**Figure 6:**
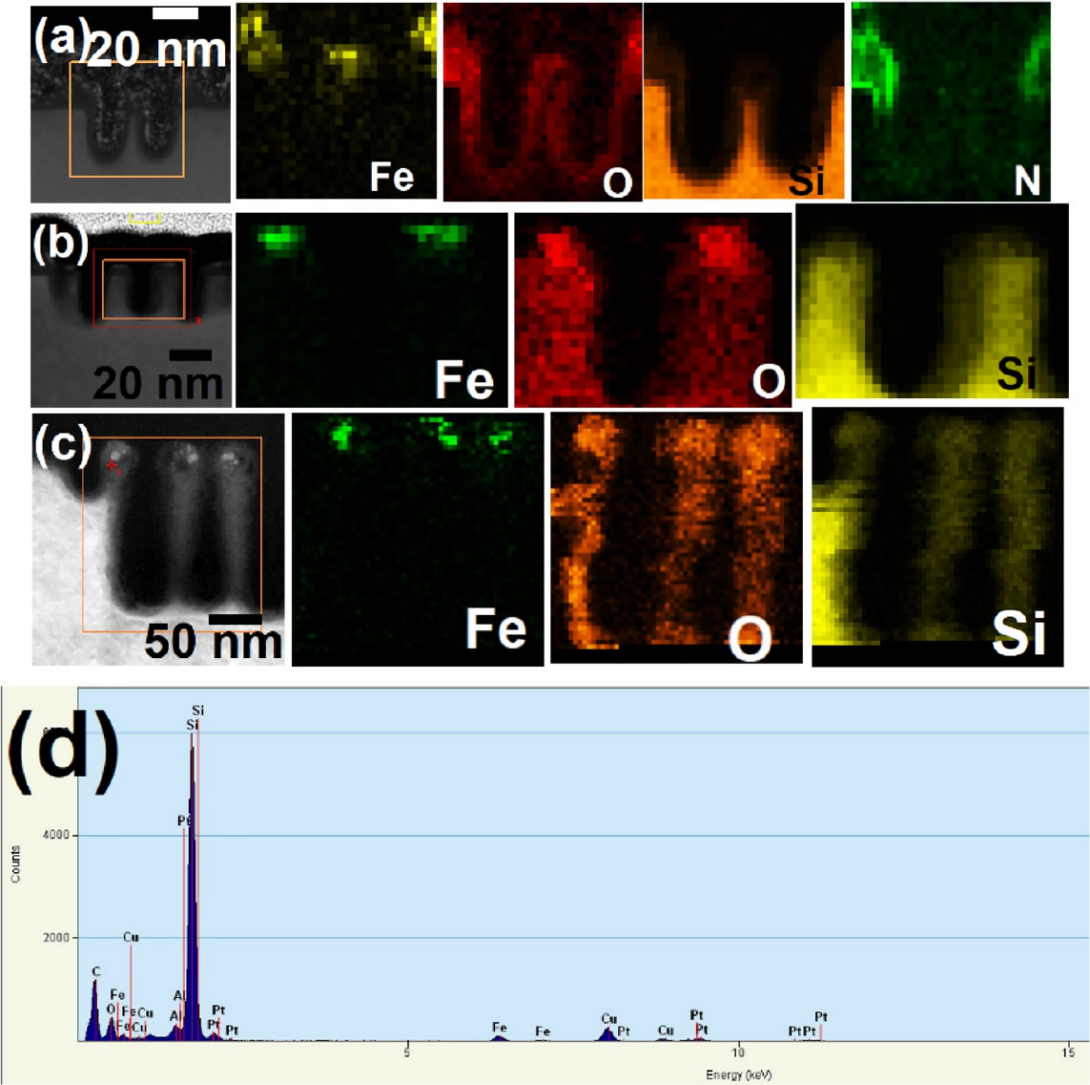
EDAX mappings of Si (a, b) 1 and 2 vertical and (c) 3 horizontal nanowire patterns with iron oxide at the top for different etch times of (a) 1 min, (b) 30 s, and (c) 1 min 30 s. (d) EDX spectrum obtained from the Si vertical nanowire arrays.

The precise structural arrangement and position of the Si, silica, Si_3_N_4_, and oxide mask were obtained from high resolution EDAX mapping. No defects or delamination of features was observed at the interface between Si nanowires and mask, reflecting the bonding and structural integrity of the mask during FIB processing. Figure 6a,b shows the elemental maps for the 1 and 2 arrays of vertical Si nanowires with a layer of silica and iron oxide at top for 60 and 30 s Si etch, respectively. The elemental composition and the distribution of Fe, O, N, and Si components is confirmed by high resolution EDAX mapping. The presence of iron confirms only at the top of the wires whereas nitrogen is at the top surface of the sidewalls. The oxygen is at the top surface of the Si nanowires and at the surface of the wires because of surface oxidation. No interdiffusion occurs. The diameter of the nanowires are ∼20 nm and they are 42 nm apart. Figure 6a,b confirms the height of the nanowires are 100 and 50 nm, respectively. Figure 6c shows 150 nm long horizontal arrays of three nanowires fabricated after 1 min 30 s etch and their elemental maps confirms sharp interfaces between Fe, O, N, and Si. EDX spectra in Figure 6d also confirms the presence of Fe, Si, and O from the nanowire arrays. All of the mapping confirms sharp elemental interfaces between the elements and no interdiffusion occurred during processing steps, which is significant from the device application perspective.

## Conclusion

Horizontal and vertical Si/SiO_2_ nanowire arrays having symmetric width throughout the entire length along the channel direction are successfully fabricated by a block-copolymer-assisted hard mask technique. A graphoepitaxial Si_3_N_4_ substrate consists of silica-coated Si channels of varying width and lengths is used to define microphase-separated PS-*b*-PEO BCP (dots and lines)/space nanopatterns. It was noticed that lower temperature solvent annealing creates hole/space patterns whereas higher temperature leads to line/space patterns irrespective of channel widths. Mixed dot and line patterns are also possible for longer annealing times. The BCP patterns are modified/etched by ethanol treatment, which is further used as a template to achieve oxide hard mask nanodots and/or nanowires arrays within trenches. Different concentrations of iron nitrate ethanolic solution is utilized to achieve monolayer, uniform diameter, continuous iron oxide nanodots, and nanowire arrays without altering the lateral spacings between them. Vertical and horizontal ordered Si nanowire structures within trenches are fabricated using iron oxide nanodots and nanowires, respectively, as a hard mask through ICP etch process after removal of the oxide mask. Cross-sectional TEM images reveal wires of uniform width and smooth sidewalls along their length, demonstrating the efficiency of the mask. Further, the aspect ratios are varied by varying etch time. The elemental maps confirm sharp interfaces between Fe, O, N, and Si without any interdiffusion. These nanowires can be functioned as a gate-all-around transistor component where the device performance can be altered by the surrounded oxide layer thickness.

## Experimental Section

### Microphase Separation of Block Copolymers Nanopatterns within Trenches

The PS-*b*-PEO BCP was purchased from Polymer Source Inc. (Canada) and used as received (number-average molecular weight, *M*_n_, PS = 42 kg mol^–1^, *M*_n_, PEO = 11.5 kg mol^–1^, the polydispersity index was 1.07). A grophoepitaxial substrate was used to generate BCP nanopatterns. Substrates were cleaned in acetone and toluene for 30 min in each solvent by ultrasonication and dried under nitrogen. PS-*b*-PEO was dissolved in toluene and aged for at least 12 h at room temperature prior to use. To achieve well-ordered long-range polymer patterns within the trenches, the polymer-solution concentrations were varied. The PS-*b*-PEO thin film was formed by spin coating (3000 rpm for 30 s). The films were placed at the bottom of a closed glass jar and exposed to toluene vapor kept at 50 and 60 °C for different time period to induce necessary chain mobility for nanopattern formation through microphase separation. Partial etching and/or modification of PEO domains was carried out by dipping the substrate at 40 °C for 16 h in anhydrous alcohol. The substrates were then removed and dried.

### Formation of Iron Oxide Nanopatterns within Trenches

For the fabrication of iron oxide nanopatterns, iron(III) nitrate nonahydrate (Fe(NO_3_)_3_·9H_2_O) was dissolved in ethanol (0.4% and 2% w/w) and spin-coated onto the substrate. UV/ozone treatment was used to oxidize the precursor and remove polymer. In this process, ozone, an active oxidizing agent, is generated *in situ* from atmospheric oxygen by exposure to 185 nm UV light. The ozone produced photodissociates into molecular oxygen and atomic oxygen successively upon exposure to 254 nm light. The latter species react with the polymer to form free radicals and activated species that eventually remove organic portions of the polymer in the form of carbon dioxide, water, and a small amount of volatile organic compounds.

### Fabrication of Si Nanopatterns within Trenches

These iron oxide nanopattern arrays were used as a hard mask to fabricate similarly ordered SiO_2_/Si nanofeatures through pattern transfer onto the underlying channels and substrate. A STS, Advanced Oxide Etch (AOE) ICP etcher was used for pattern transfer. The system has two different RF generators, one to generate and control the plasma density by direct connection to the antenna coil and another to adjust and control the energy of ions by connecting it to the substrate holder. Subsequent silica and silicon etching steps were followed to etch the silica layer and then to etch the silicon substrate. The sample is thermally attached using (Krytox) vacuum oil to a ceramic wafer that is (mechanically) clamped to the RF chuck (10 °C), and helium gas is used for backside cooling at a pressure of 9.5 Torr. For the oxide layer etch, a C_4_F_8_/H_2_ gas mixture (21 sccm/30 sccm) is used with an ICP coil power of 800 W and a Reactive Ion Etching (RIE) power of 80 W at a chamber pressure of 15 mTorr. A 10 s silica etching time was optimized for all the samples. To etch into the Si substrate, a controlled gas mixture of C_4_F_8_/SF_6_ is used at flow rates of 90 sccm/30 sccm and the ICP/RIE power of 600 W/15 W with a chamber pressure of 15 mTorr. The height of the SiO_2_/Si nanofeatures was varied by adjusting the Si etch time. The substrate was dipped into 10 wt % oxalic acid dihydrate (C_2_H_2_O_4_·2H_2_O) aqueous solution for 2 h at room temperature, washed with water several times and dried to eliminate the iron oxide mask.

### Characterizations

Surface morphologies were imaged by scanning electron microscopy (SEM, FEI Company, FEG Quanta 6700 and Zeiss Ultra Plus). Samples were prepared for transmission electron microscopy (TEM) cross sectional imaging with an FEI Helios Nanolab 600i system containing a high resolution Elstar Schottky field-emission SEM and a Sidewinder FIB column. TEM and elemental mapping were carried out on FEI Titan.

## Supporting Information Available

SEM images of the effects of low concentrations of the BCP–toluene solution on the self-assembled BCP patterns, SEM images of effects of higher concentrations of the BCP–toluene solution on the self-assembled BCP patterns, and SEM images of the BCP patterns with PS brush (PDF)
