# Supplementary material for: Fabrication of Graphoepitaxial Gate-All-Around Si Circuitry Patterned Nanowire Arrays Using Block Copolymer Assisted Hard Mask Approach
Source: ACS Nano. 2021 May 27;15(6):9550–8. doi: 10.1021/acsnano.0c09232 (PMC8291765; doi:10.1021/acsnano.0c09232)
Supplement: Supplementary file 1 — nn0c09232_si_001.pdf [file nn0c09232_si_001.pdf]

Supporting Information:

Fabrication of Graphoepitaxial Gate-all-around Si Circuitry Patterned Nanowire Arrays Using Block Copolymer Assisted Hard Mask Approach

Tandra Ghoshal,[1*] Ramsankar Senthamaraikannan,[1] Matthew T. Shaw,[2] Ross Lundy,[1] Andrew Selkirk,[1] Michael A. Morris[1*]

Effects of low concentrations of the BCP-toluene solution.

BCP concentrations in toluene were varied to achieve well-ordered nanostructures without any overfilling/missing pattern or variation in the film thickness. In order to examine the effects of BCP-toluene solution, 0.3 wt% solution was spin coated onto the substrate and further annealed in toluene at 50 °C for 1 hour. Figure S1a shows the SEM images where dots/space patterns obtained in a 90 nm wider trench in localized areas and pattern missing also evident. Similarly substrate annealed at 60 °C for 1 h forms horizontally oriented PEO microdomains inside the PS matrix where lines/space patterns are discontinues along the trench (~ 45 nm width) direction shown in Figure S1b.

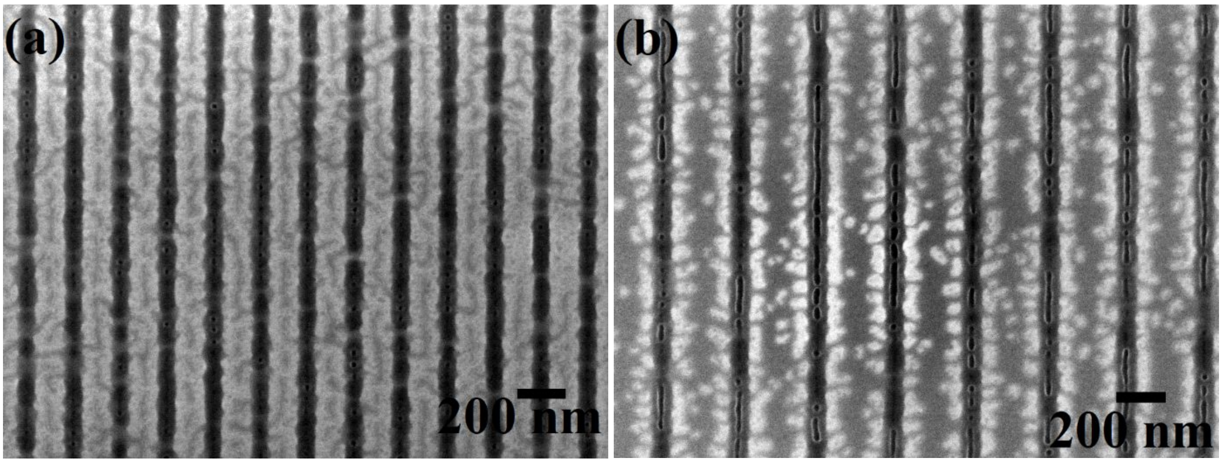

Figure S1: Dots/space patterns obtained with 0.3 wt% BCP-solution spin coated and annealed at a temperature of (a) 50 °C and (b) 60 °C for 1 h for trench widths of 90 nm and 45 nm respectively.

Effects of higher concentrations of the BCP-toluene solution.

A 0.5 wt% and 0.6 wt% BCP-toluene solution was spin coated onto the substrate and further annealed in toluene at 50 °C for 1 hour. Figure S2 shows the SEM images where dots/space patterns obtained in 90 nm and 180 nm wider trench. An overfilling of the polymer was observed all over the trenches for smaller trench width as shown in Figure S2a. Localized overfilling is noticed for wider trenches of 180 nm as shown in Figure S2b.

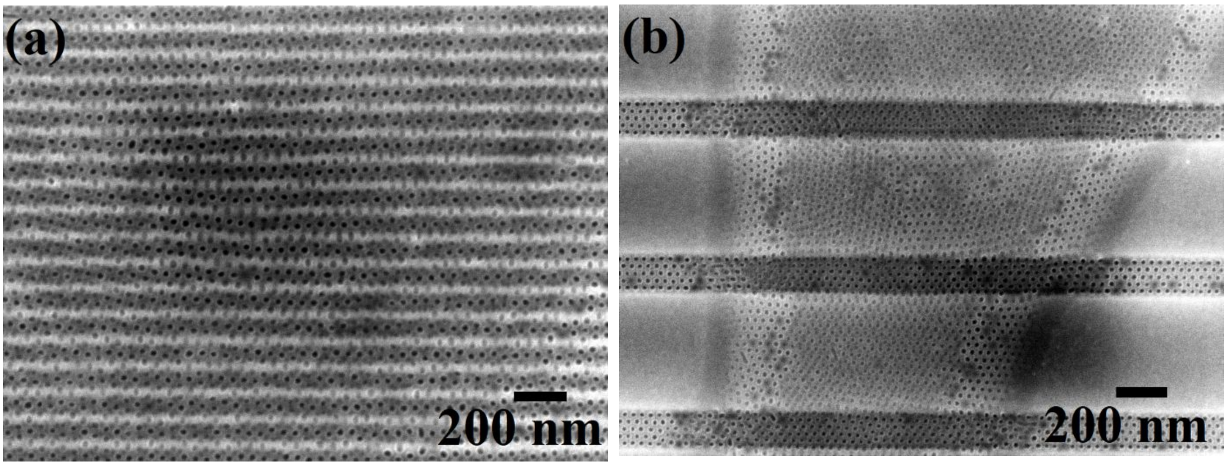

Figure S2: Dots/space patterns obtained with 0.5 wt% and 0.6 wt% BCP-solution spin coated and annealed at a temperature of 50 °C for 1 h for trench widths of (a) 90 nm and (b) 180 nm respectively.

Effects of PS brush layer on the BCP patterns.

In order to improve the BCP self-assembled nanopatterns, a neutral hydroxyl-terminated polystyrene (PS) brush (molecular weight, M_n, 10000 g/mol) layer was deposited on the trench substrate. For the brush layer attachment, we have followed the recipe by Borah et al.[1] Substrates were cleaned in a piranha solution (1:3 v/v 30% H_2O_2:H_2SO_4) at 90 °C for 60 min, rinsed with deionized water several times, and dried under N_2. The piranha activation removes

any organic contaminant and creates a high density of hydroxyl groups on the substrates. Polymer brush solution (1.0 wt % in toluene) was spin-coated onto the substrates at 3000 rpm for 30 s. This was annealed in a vacuum oven (Townson and Mercer EV018) at 170 ℃ under vacuum for 6 h. This procedure provides chemically anchored brush by condensation reactions between −OH groups at the substrate surface and on the brush. Unbound polymers were removed by sonication (Cole-Palmer Model 8891 sonicator) and rinsing in toluene. This results a 7.3 nm brush layer within the trenches.

Figure S3 shows SEM images of solvent annealed PS-*b*-PEO thin films annealed at 50 °C for 1 hour forming hexagonally packed PEO microdomains perpendicular to the substrate surface with a centre-to-centre spacing of 42 nm. 2 and 4 arrays of long range ordered perpendicularly oriented PEO microdomains inside the PS matrix were realized in wafer scale for the channel width of 90 nm and 180 nm respectively. The structures were almost similar as obtained without the brush layer.

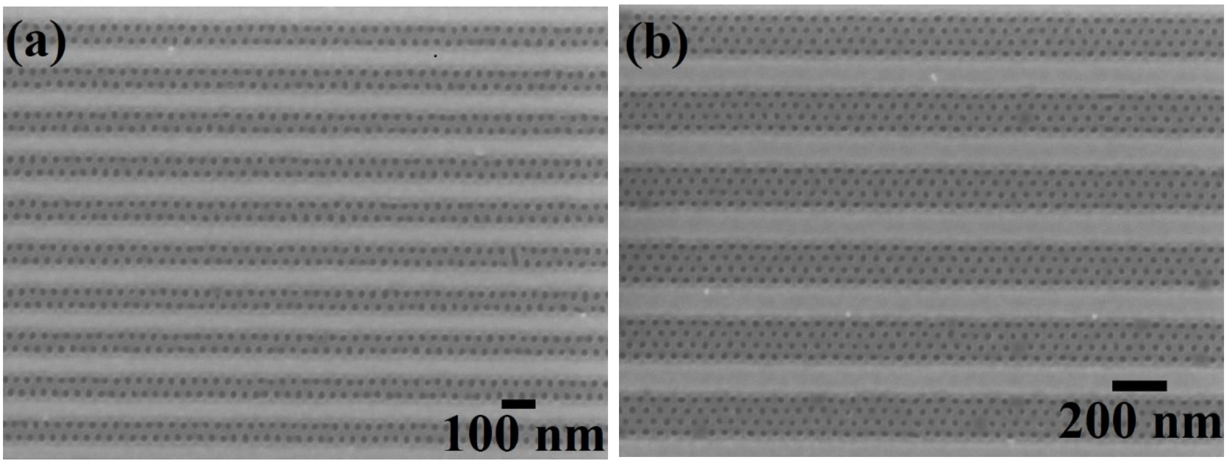

Figure S3: Dots/space patterns obtained with PS brush layer with 0.4 wt% BCP-solution spin coated and annealed at a temperature of 50 °C for 1 h for trench widths of (a) 90 nm and (b) 180 nm respectively.

At an elevated solvent annealed temperature of 60 °C, the BCP was overfilled in few places. Perpendicularly oriented BCP patterns were realized in most of the places whereas overfilled trench part gives parallel orientation of PEO cylinders as shown in Figure S4.

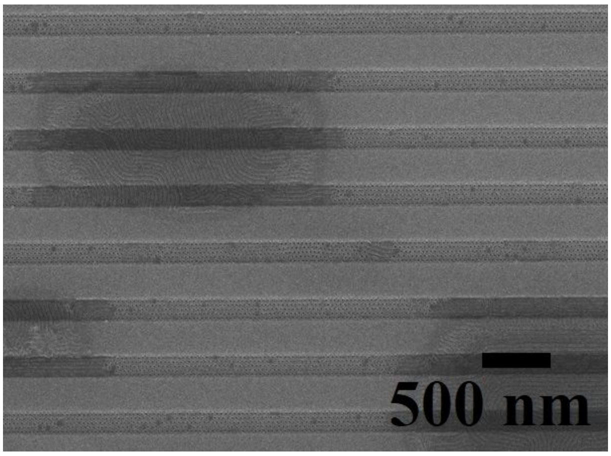

Figure S4: Patterns obtained with PS brush layer with 0.4 wt% BCP-solution spin coated and annealed at a temperature of 60 °C for 1 h.

References:

(1) Borah, D.; Rasappa, S.; Senthamaraikannan, R.; Kosmala, B.; Shaw, M. T.; Holmes, J. D.; Morris, M. A. Orientation and Alignment Control of Microphase-Separated PS-b-PDMS Substrate Patterns *via* Polymer Brush Chemistry. *ACS Appl. Mater. Interfaces* **2013**, 5, 88-97.
